# Statin-induced expression of CD59 on vascular endothelium in hypoxia: a potential mechanism for the anti-inflammatory actions of statins in rheumatoid arthritis

**DOI:** 10.1186/ar2019

**Published:** 2006-07-21

**Authors:** Anne R Kinderlerer, Rivka Steinberg, Michael Johns, Sarah K Harten, Elaine A Lidington, Dorian O Haskard, Patrick H Maxwell, Justin C Mason

**Affiliations:** 1 Cardiovascular Medicine Unit, Eric Bywaters Center for Vascular Inflammation, Imperial College London, Hammersmith Hospital, London, UK; 2 The Renal Unit, Imperial College London, Hammersmith Hospital, London, UK

## Abstract

Hypoxia, which leads to dysfunctional cell metabolism, and complement activation both play central roles in the pathogenesis of rheumatoid arthritis (RA). Recent studies have reported that mice deficient for the complement-inhibitory protein CD59 show enhanced susceptibility to antigen-induced arthritis and reported that statins have anti-inflammatory effects in RA. We hypothesized that the anti-inflammatory effect of statins in RA relates in part to their ability to increase CD59 expression in hypoxic conditions and therefore to reduce complement activation.

Flow-cytometric analysis showed that CD59 expression on endothelial cells (EC) was unaffected by atorvastatin in normoxia (21% O_2_), whereas in hypoxic conditions (1% O_2_) an up to threefold dose-dependent increase in CD59 expression was seen. This effect of hypoxia was confirmed by treatment of EC with chemical mimetics of hypoxia. The upregulation of CD59 protein expression in hypoxia was associated with an increase in steady-state mRNA. L-Mevalonate and geranylgeraniol reversed the response, confirming a role for inhibition of 3-hydroxy-3-methylglutaryl coenzyme A reductase and geranylgeranylation. Likewise, inhibition by *N*^G^-monomethyl-L-arginine and *N*^G^-nitro-L-arginine methyl ester confirmed that CD59 upregulation in hypoxia was nitric oxide dependent. The expression of another complement-inhibitory protein, decay-accelerating factor (DAF), is known to be increased by atorvastatin in normoxia; this response was also significantly enhanced under hypoxic conditions. The upregulation of CD59 and DAF by atorvastatin in hypoxia prevented the deposition of C3, C9 and cell lysis that follows exposure of reoxygenated EC to serum. This cytoprotective effect was abrogated by inhibitory anti-CD59 and anti-DAF mAbs. The modulation of EC CD59 and DAF by statins under hypoxic conditions therefore inhibits both early and late complement activation and may contribute to the anti-inflammatory effects of statins in RA.

## Introduction

Analysis of the rheumatoid joint reveals it to be a hypoxic environment with mean intra-articular PO_2 _values as low as 13 mmHg [1,2]. This reflects in part the influence of synovial cell proliferation and increased metabolic demand. In addition, despite increased angiogenesis, the location of capillaries deep within the synovium and the relatively reduced capillary density result in inadequate tissue perfusion [3]. This is further exacerbated by movement, which increases the intra-articular pressure and results in periodic microvessel occlusion and cycles of hypoxia–reoxygenation [2]. The latter leads to chronic oxidative stress, to generation of reactive oxygen species [1,2] and to enhanced expression of proinflammatory mediators including cyclooxygenase-2-derived nociceptive eicosanoids and matrix metalloproteinases [4,5]. Hypoxic conditions within the rheumatoid joint induce expression of the principal regulator of the adaptive response to hypoxia, hypoxia-inducible factor (HIF). The HIF-1α and HIF-2α levels are increased in synovial fibroblasts, macrophages and endothelial cells (EC) [6], and HIF-1α expression has been identified in the lining and sublining layer of rheumatoid synovium [7].

Increased levels of complement activation products are present in the synovium, serum and synovial fluid of rheumatoid arthritis (RA) patients and correlate with disease activity [8,9]. Deposition of C3 and the C5b-9 membrane attack complex (MAC) has been demonstrated in the synovial lining layer and on EC in the synovium and rheumatoid nodules [10-12]. Potential triggers for complement activation include rheumatoid factor immune complexes and C-reactive protein [8]. Furthermore, exposure of EC to prolonged hypoxia and reoxygenation also results in complement activation [13], which may represent an additional means by which the complement cascade is activated in the rheumatoid joint.

Deposition of the MAC may exert proinflammatory effects, proproliferative effects and proapoptotic effects on synovial cells and EC, and may modulate leukocyte recruitment [14]. The MAC induces prostaglandin E_2 _release from rheumatoid synovial cells [15]. Proinflammatory actions on EC are mediated through activation of NF-κB, through induction of E-selectin and intercellular adhesion molecule-1 expression [16], and through release of chemokines including monocyte chemoattractant protein-1 and IL-8 [14,17].

The membrane-bound complement regulatory proteins decay-accelerating factor (DAF, CD55), membrane cofactor protein (MCP, CD46), complement receptor-1 and CD59 provide protection from autologous complement-mediated injury [18]. DAF and MCP act at the level of the C3 convertase. In contrast, CD59 inhibits the terminal pathway of complement activation, preventing the incorporation of C9 into the MAC [18]. While DAF expression is increased in the rheumatoid synovium [10], expression of CD59 is significantly decreased on the synovial lining, stromal cells and EC [11]. Moreover, injection into the rat knee joint of an anti-rat CD59 mAb induces a spontaneous complement-dependent arthritis [19], and CD59-deficient mice are prone to enhanced antigen-induced arthritis [20].

We have previously reported that, under normoxic conditions, statins (3-hydroxy-3-methylglutaryl coenzyme A (HMG-CoA) reductase inhibitors) significantly upregulate DAF expression but not CD59 expression on EC, resulting in protection against complement-mediated injury [21]. *In vitro *experiments have revealed, however, that the effect of statins on endothelial function may be enhanced by hypoxia [22]. Furthermore, two of three recent studies have demonstrated clinically apparent anti-inflammatory effects of statins in rodent models of inflammatory arthritis and in one model in patients with RA [23-26]. These findings led us to explore the hypothesis that, under prolonged hypoxic conditions such as those present in the rheumatoid joint, statins are able to enhance expression of CD59, so minimizing generation of the C5b-9 MAC and its proinflammatory consequences. Vascular EC represented a cell type on which to test this hypothesis, because the endothelium is exposed to hypoxia, as evidenced by expression of HIF-1α [6], and represents a major site of complement deposition in the rheumatoid joint [9].

In the present study, we show for the first time that statins can upregulate CD59 on EC in hypoxia and that hypoxic conditions also enhance statin-induced DAF induction. These combined effects result in significantly enhanced protection against complement activation and may represent an important novel contributory mechanism to the anti-inflammatory effects of statins in RA.

## Materials and methods

### Monoclonal antibodies and other reagents

CD59 mAb (IgG_1_) Bric 229 was purchased from the International Blood Group Reference Laboratory (Bristol, UK). Anti-DAF mAb 1H4 (IgG_1_) and anti-MCP mAb TRA-2-10 (IgG_1_) were gifts from D Lublin and J Atkinson, respectively (Washington University School of Medicine, St Louis, MO, USA). Atorvastatin and lovastatin were from Merck Biosciences Ltd (Nottingham, UK). Lovastatin was chemically activated before use by alkaline hydrolysis. Pre-activated mevastatin, *N*^G^-monomethyl-L-arginine (L-NMMA), *N*^G^-nitro-L-arginine methyl ester (L-NAME) and geranylgeraniol were from BIOMOL (Plymouth Meeting, PA, USA). Other products were obtained from Sigma (Poole, UK). In all experiments, EC were also treated with the appropriate drug vehicle controls.

### Endothelial cell isolation and culture

Human umbilical vein endothelial cells (HUVEC) were isolated and cultured as described previously [27]. For hypoxia experiments, confluent monolayers in tissue culture plates were cultured in a hypoxic gas mixture consisting of 1% O_2_, 94% N_2 _and 5% CO_2 _in a Galaxy Rincubator (Wolf Laboratories, York, UK) or in a hypoxic chamber with gloveport access (Ruskinn Technologies, Cincinnati, OH, USA). The chemical mimetics of hypoxia, cobalt chloride (CoCl_2_) and desferrioxamine (DFO) (both from Sigma), were added to EC cultures 30 minutes prior to the addition of atorvastatin and remained throughout the experiment. Our human tissue protocols were approved by the hospital Research Ethics Committee.

### Flow cytometry

Flow cytometry was performed as described previously [27]. Pharmacological antagonists were added 60 minutes before the addition of statins. In some experiments the results are expressed as the relative fluorescence intensity (RFI), representing the mean fluorescence intensity with test mAb divided by the mean fluorescence intensity using an isotype-matched irrelevant mAb. Cell viability was assessed by examination of EC monolayers using phase-contrast microscopy, cell counting and estimation of trypan blue exclusion.

### Western blotting

HUVEC were lysed in urea–sodium dodecyl sulphate buffer (6.7 M urea, 10 mM Tris–Cl (pH 6.8), 1 mM dithiothreitol, 10% glycerol, 1% sodium dodecyl sulphate). Extracts were normalized for protein content, were resolved by SDS-PAGE and were transferred onto polyvinylidene difluoride membrane. Blots were probed with mouse mAbs against HIF-1α (54), HIF-2α (190b) (Transduction Labs, Lexington, KY, USA) and α-tubulin (Sigma), followed by a horseradish peroxidase-conjugated secondary anti-mouse antibody (DAKO, Ely, UK) and detection with the ECL Plus system (Amersham Biosciences, Little Chalfont, UK).

### Northern blotting and real-time PCR

HUVEC were exposed to 1% O_2 _and treated with atorvastatin for the indicated times and then RNA was extracted using the RNeasy kit (Qiagen Ltd, Crawley, UK). Total RNA was separated on a 1% agarose/formaldehyde gel, transferred overnight to Hybond-N nylon membranes (Amersham Biosciences) and was analysed by specific hybridization to a radiolabelled cDNA probe for human CD59 (gift from H Waldmann, University of Oxford, UK) as described previously [27]. Integrated density values for each band were obtained with an Alpha Innotech ChemiImager 5500 (Alpha Innotech, San Leandro, CA, USA), normalized with respect to the 28S band on ethidium bromide-stained rRNA loading patterns and expressed as the percentage change from control.

Quantitative real-time PCR was carried out using an iCycler (BioRad, Hercules, CA, USA). DNase-1-digested total RNA (1 μg) was reverse transcribed using 1 μM oligo-dT and Superscript reverse transcriptase (Invitrogen, Paisley, UK), according to the manufacturers' instructions. For measurement of CD59 and β-actin, cDNA was amplified in a 25 μl reaction containing 5 μl cDNA template, 12.5 μl iSYBR supermix (BioRad), and 0.5 pmol each sense and 0.5 pmol each antisense gene-specific primer. The volume was adjusted to 25 μl with ddH_2_O. The primer sequences used were as follows: β-actin forward, GAGCTACGAGCTGCCTGACG; β-actin reverse, GTAGTTTCGTGGATGCCACAGGACT; CD59 forward, ATGCGTGTCTCATTAC; and CD59 reverse TTCTCTGATAAGGATGTC. The cycling parameters were 3 minutes at 95°C followed by 40 cycles of 95°C for 10 seconds and of 56°C for 45 seconds. In experiments designed to assess the mRNA stability, EC were pretreated with actinomycin D (2 μg/ml).

### Complement deposition and lysis assays

Cell surface C3 and C9 deposition was assessed by flow cytometry. HUVEC were incubated in 1% or 21% O_2 _for 48 hours with or without atorvastatin and were then reoxygenated for 3 hours. For analysis of the C3 binding, EC were suspended in veronal buffered saline containing 0.1% gelatin (VBSG), in 20% C5-deficient serum (Sigma) or in 20% heat-inactivated serum. For analysis of C9 deposition, cells were resuspended in VBSG, in 20% normal human serum or in heat-inactivated serum. EC were incubated with serum for 90 minutes (C3 binding) and for 2 hours (C9 binding) at 37°C. Flow-cytometric assessment of C3 binding was detected with fluorescein isothiocyanate-conjugated anti-C3 (1:40; DAKO), and C9 binding was assessed with mouse anti-human C5b-C9 (Technoclone, Vienna, Austria).

Complement-mediated cell lysis was measured by assessing the percentage of cells permeable to propidium iodide using flow cytometry, following exposure to rabbit serum (Serotec, Oxford, UK). HUVEC exposed to the same conditions as for C9 binding were treated with the inhibitory mAbs 1H4 [28] and Bric 229 [29] (20 μg/ml) in VBSG. These HUVEC were then incubated with VBSG, with 20% rabbit serum or with heat-inactivated rabbit serum for 90 minutes at 37°C. The cells were then washed and propidium iodide (final concentration 50 μg/ml) was added. The percentage of cells positive for propidium iodide was measured in the FL2 channel on a Beckman-Coulter flow cytometer (Luton, UK).

### Statistical analysis

All data were expressed as the mean of the individual experiments ± the standard error of the mean. Data were analysed using one-way or two-way analysis of variance with Bonferroni correction. Normalized data were analysed using the Wilcoxon Rank Sum test (GraphPad Prism Software, San Diego, CA, USA). Differences were considered significant at *P *< 0.05.

## Results

### Atorvastatin induces CD59 expression on endothelial cells in hypoxia

Previous studies have demonstrated that statins and hypoxia may act both independently and synergistically to induce cytoprotective pathways in vascular EC [30]. To explore the effect of atorvastatin on EC CD59 expression in hypoxia, we cultured HUVEC in 1% oxygen. We have previously demonstrated that expression of CD59 on the surface of HUVEC is directly comparable with that on the surface of microvascular and arterial EC [21,27]. Cultured EC are typically maintained at a partial pressure of oxygen of 154 mmHg (21% O_2_) (at atmospheric pressure), whereas *in vivo *EC are exposed to a partial pressure of oxygen of 20–25 mmHg (3–5% O_2_) – culture in 1% O_2 _(8 mmHg) therefore represents true hypoxia when compared to normoxic levels of 3–5% O_2_* in vivo*.

**Figure 1:**
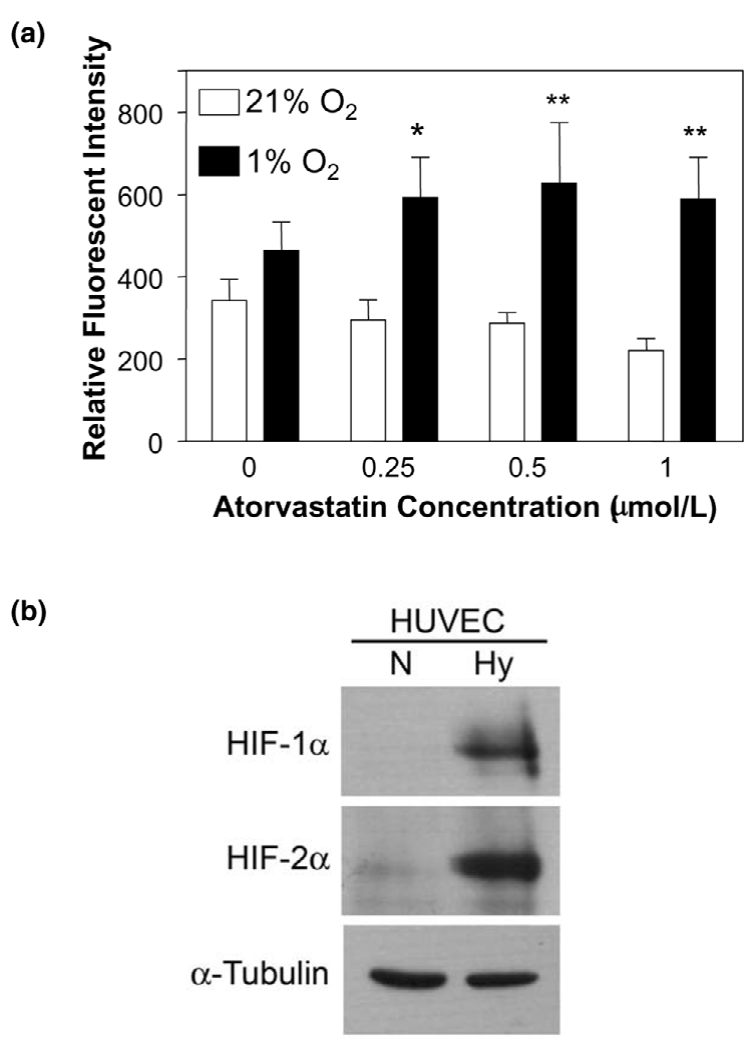
Atorvastatin enhances CD59 expression in hypoxia on endothelial cells. **(a) **Following culture for 48 hours in 21% O_2 _(normoxia, open bars) or 1% O_2 _(hypoxia, filled bars), in the presence of increasing concentrations of atorvastatin, endothelial cell CD59 expression was measured by flow cytometry using the mAb BRIC 229. Bars represent the mean relative fluorescence intensity ± standard error of the mean, derived by dividing the mean fluorescence intensity obtained with test mAb by the mean fluorescence intensity with irrelevant isotype-matched control mAb (*n *= 4), *P < 0.05, **P < 0.01 compared with untreated controls. **(b) **Human umbilical vein endothelial cells (HUVEC) cultured for 24 hours in 21% O_2 _(normoxia, N) or 1% O_2 _(hypoxia, Hy) were lysed and analysed by immunoblotting for expression of HIF-1α, HIF-2α and α-tubulin as a loading control.

**Figure 2:**
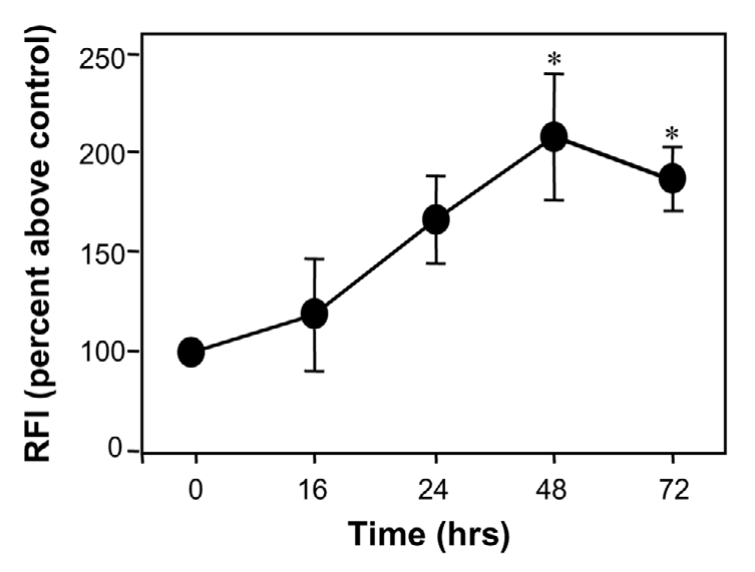
Kinetics for the upregulation of CD59 by atorvastatin. Endothelial cells were treated with atorvastatin (0.5 μM) for up to 72 hours in hypoxic conditions (1% O_2_) prior to flow-cytometric analysis of CD59 expression using the mAb BRIC 229. Results are expressed as the percentage increase in relative fluorescence intensity (RFI) above the unstimulated control ± standard error of the mean (*n *= 3), *P < 0.05.

As seen in Figure 1, neither exposure to hypoxia nor treatment with atorvastatin alone significantly affected CD59 expression. Although on occasion atorvastatin alone led to a decrease in CD59 expression, this did not reach significance. Treatment with atorvastatin at concentrations up to 1 μM for 48 hours under hypoxic conditions, however, resulted in a dose-dependent increase in CD59 expression (Figure 1a). Treatment with atorvastatin in hypoxia increased the RFI for CD59 from 287.4 ± 25.5 to 627.69 ± 147.1 (*P *< 0.01). The efficacy of the hypoxic environment used was confirmed by the induction of HIF-1α and HIF-2α expression in EC following 24 hours of culture under these conditions (Figure 1b). The increase in expression of CD59 was first detectable at 16 hours, was maximal at 48 hours and was sustained at 72 hours post-treatment (*P *< 0.05) (Figure 2).

Further experiments performed under hypoxic conditions showed that both mevastatin and lovastatin increased CD59 expression to a similar degree to atorvastatin (data not shown), suggesting this is a statin class effect.

### Chemical mimics of hypoxia enhance atorvastatin-induced CD59 expression

We sought to confirm the effect of hypoxia on statin-induced CD59 expression using CoCl_2 _and DFO. These compounds mimic hypoxia through competition for and chelation of free iron, respectively, stabilizing HIF-1α under normoxic conditions [31].

**Figure 3:**
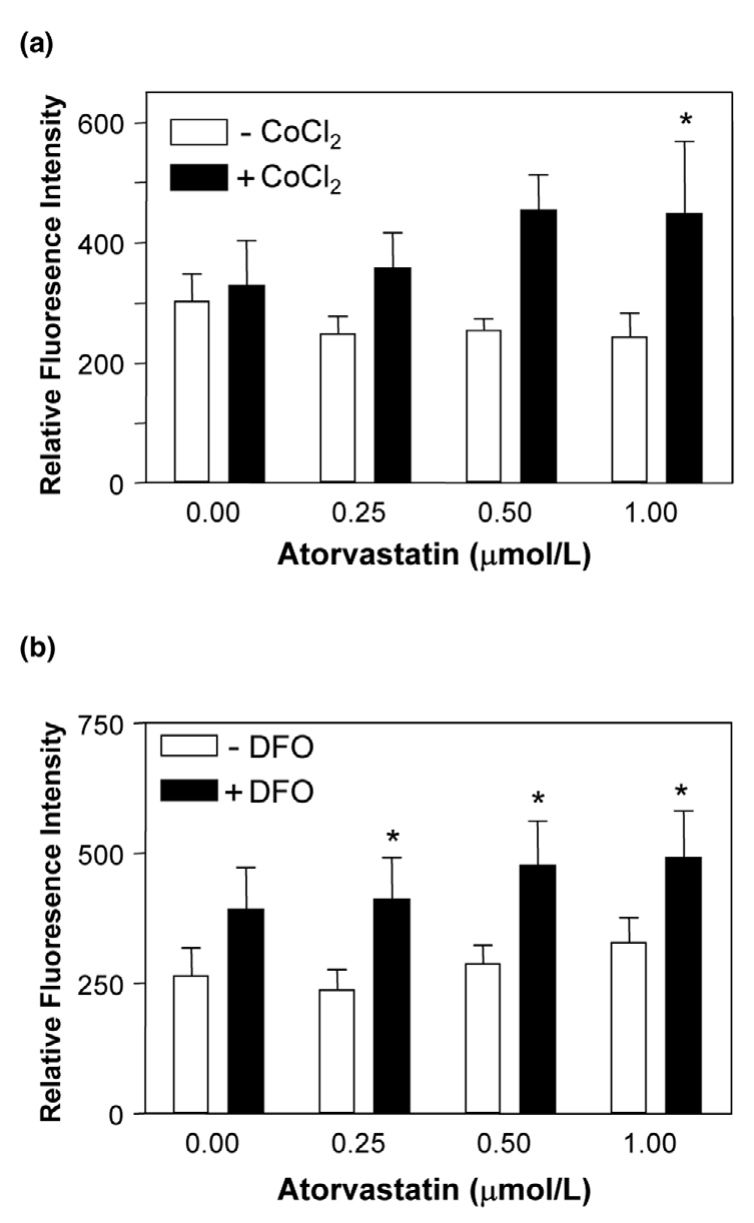
Effect of chemical mimetics of hypoxia on CD59 expression. HUVEC were treated with increasing concentrations of atorvastatin for 48 hours in the presence (filled bars) or absence (open bars) of **(a) **cobalt chloride (CoCl_2_) (100 μM) or **(b) **desferrioxamine (DFO) (100 μM). Endothelial cell CD59 expression was measured by flow cytometry using the mAb BRIC 229. Bars represent the mean ± standard error of the mean relative fluorescence intensity (*n *= 4). *P < 0.05, **P < 0.01 compared with untreated control.

CoCl_2 _alone had no effect on CD59 expression (Figure 3a), whereas DFO increased expression by 50% (Figure 3b). When EC were treated with atorvastatin in combination with either CoCl_2 _or DFO, we observed a significant increase in CD59; following 48 hours of treatment with atorvastatin + CoCl_2 _or with atorvastatin + DFO there was an up to twofold increase in cell surface CD59 (*P *< 0.05) (Figure 3). These data further support a permissive role for hypoxia in statin-induced CD59 expression.

### CD59 mRNA is increased by exposure to hypoxia and atorvastatin

**Figure 4:**
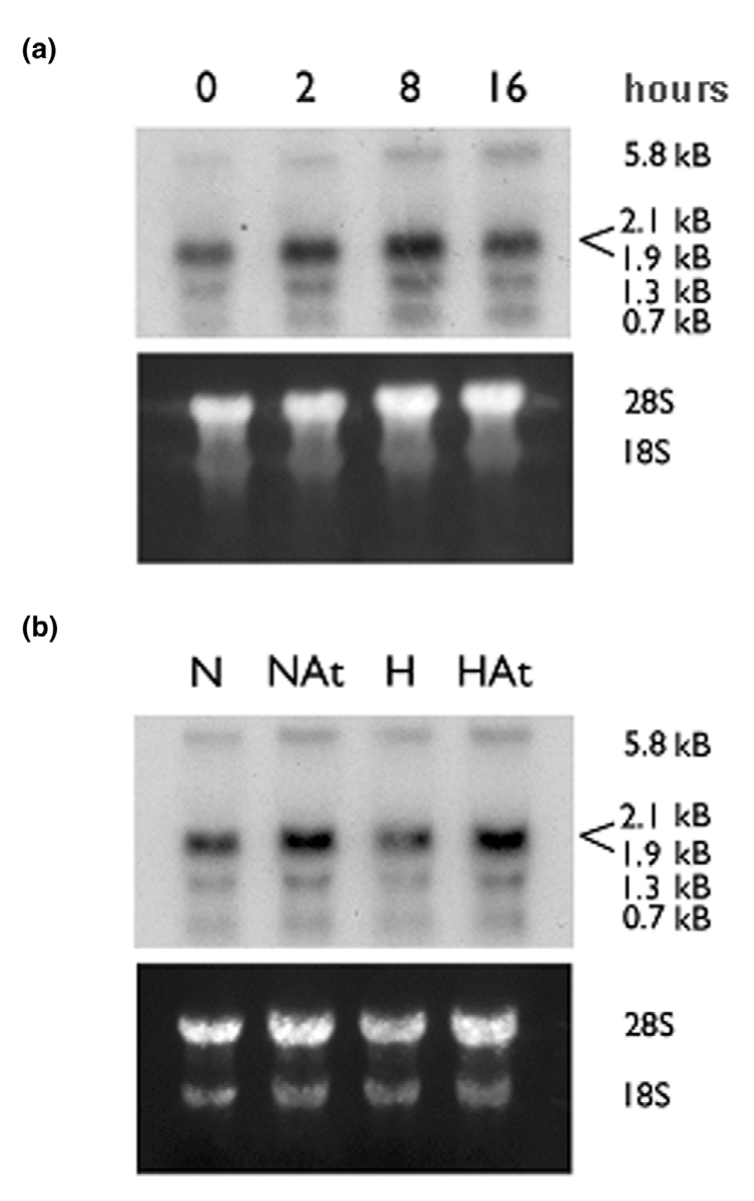
Atorvastatin increases CD59 mRNA levels in endothelial cells. **(a) **Human umbilical vein endothelial cells (HUVEC) were treated with atorvastatin (0.5 μM) and cultured for up to 16 hours in hypoxic conditions (1% O_2_). **(b) **HUVEC were cultured in normoxic (N) or hypoxic (H) conditions for 8 hours in the presence (At) or absence of atorvastatin 0.25 μM. Total RNA was isolated, and northern blots were prepared and probed for CD59 mRNA.

Northern analysis was performed to determine whether the change in CD59 expression involved gene transcription. mRNA was extracted from unstimulated and atorvastatin-treated HUVEC following culture under normoxic conditions and under hypoxic (1% O_2_) conditions for up to 16 hours. Northern analysis revealed multiple CD59 splice variants in untreated EC (0 hours; Figure 4a). Quantification of mRNA using the 2.1 kB band indicated a mean ± standard deviation increase of 54 ± 17% after 8 hours of stimulation with atorvastatin in hypoxia, which had returned to baseline at 16 hours.

A comparison of the effect of atorvastatin treatment of EC for 8 hours in normoxic conditions and in hypoxic conditions suggested that, even under normoxic conditions, steady-state CD59 mRNA levels were increased by 20% by treatment with atorvastatin (Figure 4b). Further experiments using quantitative real-time PCR produced similar results with a mean ± standard deviation increase of 105 ± 4.3% in CD59 mRNA following an 8-hour treatment with atorvastatin in hypoxia. Likewise, atorvastatin treatment in normoxic conditions induced a 22 ± 12.5% increase in CD59 mRNA.

In light of the fact that hypoxia shortens the half-life of endothelial nitric oxide (NO) synthase mRNA and that simvastatin exerts a stabilizing effect [32], we performed quantitative real-time PCR analysis in the presence of actinomycin D. In contrast to endothelial NO synthase, hypoxia did not reduce the CD59 mRNA half-life, and treatment with atorvastatin in both hypoxia and normoxia had no significant effect on CD59 mRNA stability (data not shown).

### Effect of mevalonate and isoprenoid intermediates

**Figure 5:**
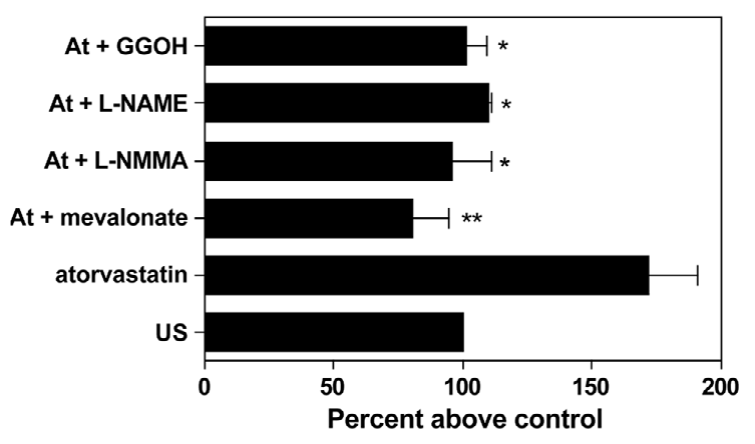
Mechanisms involved in atorvastatin-induced decay-accelerating factor expression. Human umbilical vein endothelial cells (HUVEC) were cultured for 48 hours under hypoxia (1% O_2_) and were treated with atorvastatin (At) (0.5 μM) in the presence or absence of mevalonate (200 μM), *N*^G^-monomethyl-L-arginine (L-NMMA) (500 μM), *N*^G^-nitro-L-arginine methyl ester (L-NAME) (100 μM) and geranylgeraniol (GGOH) (20 μM). Endothelial cell CD59 expression was measured by flow cytometry using the mAb BRIC 229. Results are expressed as the percentage increase in relative fluorescence intensity above the hypoxic control (US) (*n *= 4). *P < 0.5, **P < 0.01 compared with untreated controls.

To confirm that changes in CD59 expression following treatment with statins under hypoxic conditions were a specific response to the inhibition of HMG-CoA reductase, HUVEC were pretreated with L-mevalonic acid, which completely inhibited the upregulation of CD59 (*P *< 0.01) (Figure 5). In light of reports that statins [33] and hypoxia [34] may increase EC NO synthesis, the effects of the NO synthase inhibitors L-NMMA and L-NAME were examined. As seen in Figure 5, the presence of either L-NMMA or L-NAME significantly reduced the upregulation by atorvastatin and the hypoxia of CD59 (*P *< 0.05).

Analysis of the effects of statins on NO bioavailability has suggested that the isoprenoid intermediates geranylgeranyl pyrophosphate and geranylgeraniol, but not farnesyl pyrophosphate or squalene, reverse statin-mediated effects. The role of geranylgeranylation in CD59 expression was therefore examined. The presence of geranylgeraniol inhibited the upregulation of CD59 to a similar degree to L-NMMA (*P *< 0.05) (Figure 5). To confirm that effects on CD59 expression were lipid independent, EC were pretreated with the cholesterol precursor squalene and this had no effect on the response (data not shown). The concentrations of the mevalonate pathway intermediates used have been established in previous work [21].

### Hypoxia enhances statin-induced decay-accelerating factor expression

We have previously reported that in normoxic conditions atorvastatin and simvastatin upregulate the expression of the complement-inhibitory protein (CIP) DAF on EC, and that this, by acting at the level of the C3 convertase, inhibits complement activation on the cell surface [21]. In light of the permissive influence of hypoxia on atorvastatin-induced CD59 expression, we sought to establish whether hypoxic conditions increased atorvastatin-induced DAF expression.

**Figure 6:**
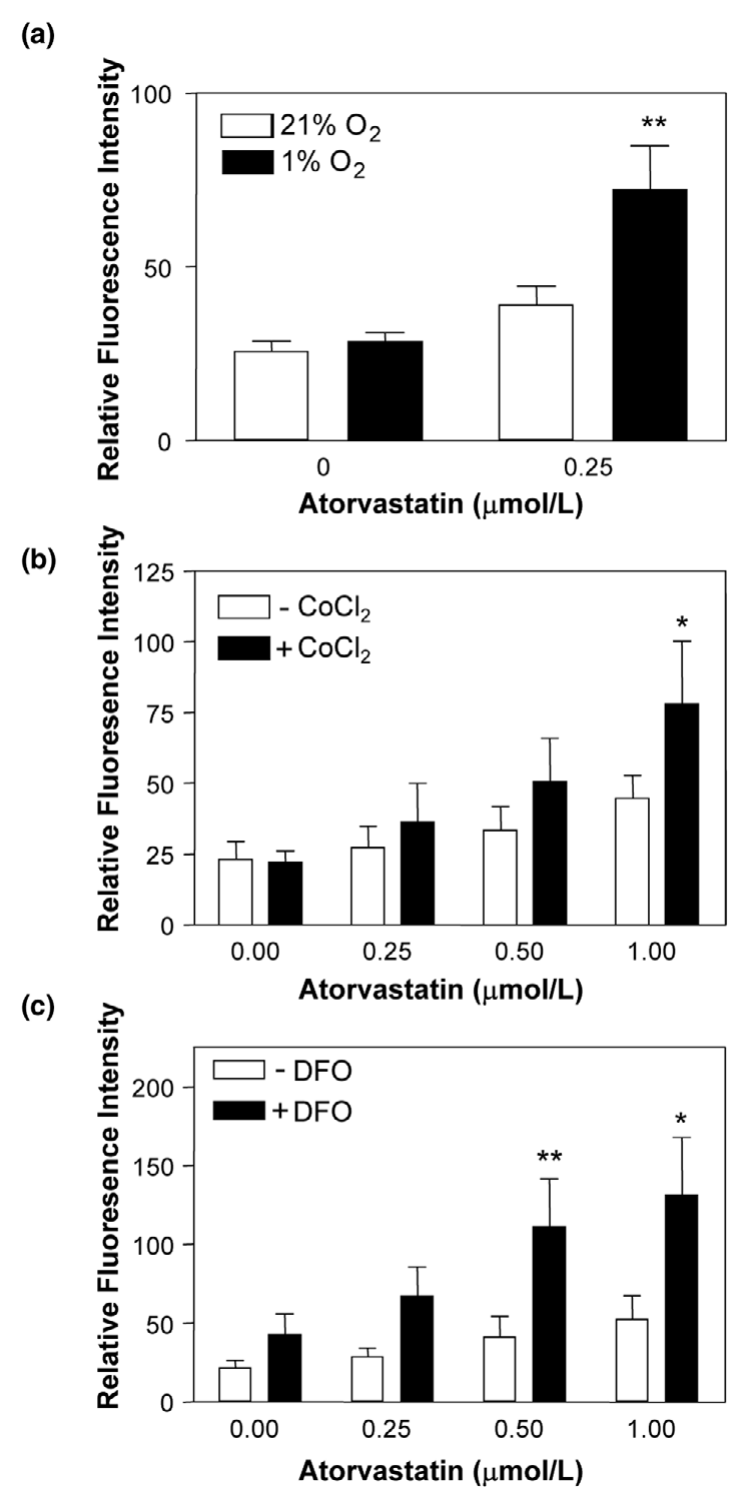
Hypoxia increases atorvastatin-induced decay-accelerating factor expression. Analysis of decay-accelerating factor expression on human umbilical vein endothelial cells (HUVEC) **(a) **following 48 hours culture in 21% O_2 _(open bars) or 1% O_2 _(filled bars) in the presence or absence of atorvastatin (0.25 μM). **(b) **and **(c) **HUVEC were treated with increasing concentrations of atorvastatin for 48 hours in the presence (filled bars) or absence (open bars) of (b) cobalt chloride (CoCl_2_) (100 μM) or (c) desferrioxamine (DFO) (100 μM). Decay-accelerating factor expression was measured by flow cytometry using the mAb 1H4. Bars represent the mean ± standard error of the mean (*n *= 4). *P < 0.05, **P < 0.01 compared with untreated controls.

HUVEC were treated with 0.25 μM atorvastatin for up to 48 hours, a concentration determined by previous studies to have a suboptimal effect on DAF expression in normoxia [21] (Figure 6a). In our hands, DAF expression was not significantly increased following exposure to 1% O_2 _for up to 48 hours (Figure 6a). Analysis of EC treated with 0.25 μM atorvastatin under hypoxic conditions for 48 hours, however, demonstrated a significant increase in DAF expression compared with that seen under normoxic conditions (Figure 6a) OK. DAF expression was increased to a similar degree under hypoxic conditions by mevastatin and lovastatin (data not shown), suggesting this is a statin class effect. MCP expression was also increased by 48 hours of culture in hypoxic conditions, as previously reported [13], although the expression was unaffected by statins (data not shown).

Further experiments using the chemical mimetics of hypoxia demonstrated that CoCl_2 _alone had no effect on DAF expression (Figure 6b), whereas DFO increased expression up to twofold (Figure 6c). When EC were treated with atorvastatin in combination with either CoCl_2 _or DFO, a significant increase in DAF expression was observed; following 48 hours of treatment with atorvastatin + CoCl_2_, the RFI ± standard error of the mean increased from 26.6 ± 7.4 to 47.7 ± 10.5 (*P *< 0.05) (Figure 6b). Treatment of EC with atorvastatin and DFO resulted in a sevenfold increase in DAF expression (mean RFI ± standard error of the mean, 21.9 ± 4.5 on unstimulated cells and 131.9 ± 36.1 on EC treated with atorvastatin and DFO) (*P *< 0.001) (Figure 6c). The enhanced effect of statins on EC CD59 and DAF expression in hypoxia are further examples of the permissive effect of hypoxia on the vasculoprotective effect of statins [30].

### Statin-induced decay-accelerating factor and CD59 expression in hypoxia is cytoprotective

**Figure 7:**
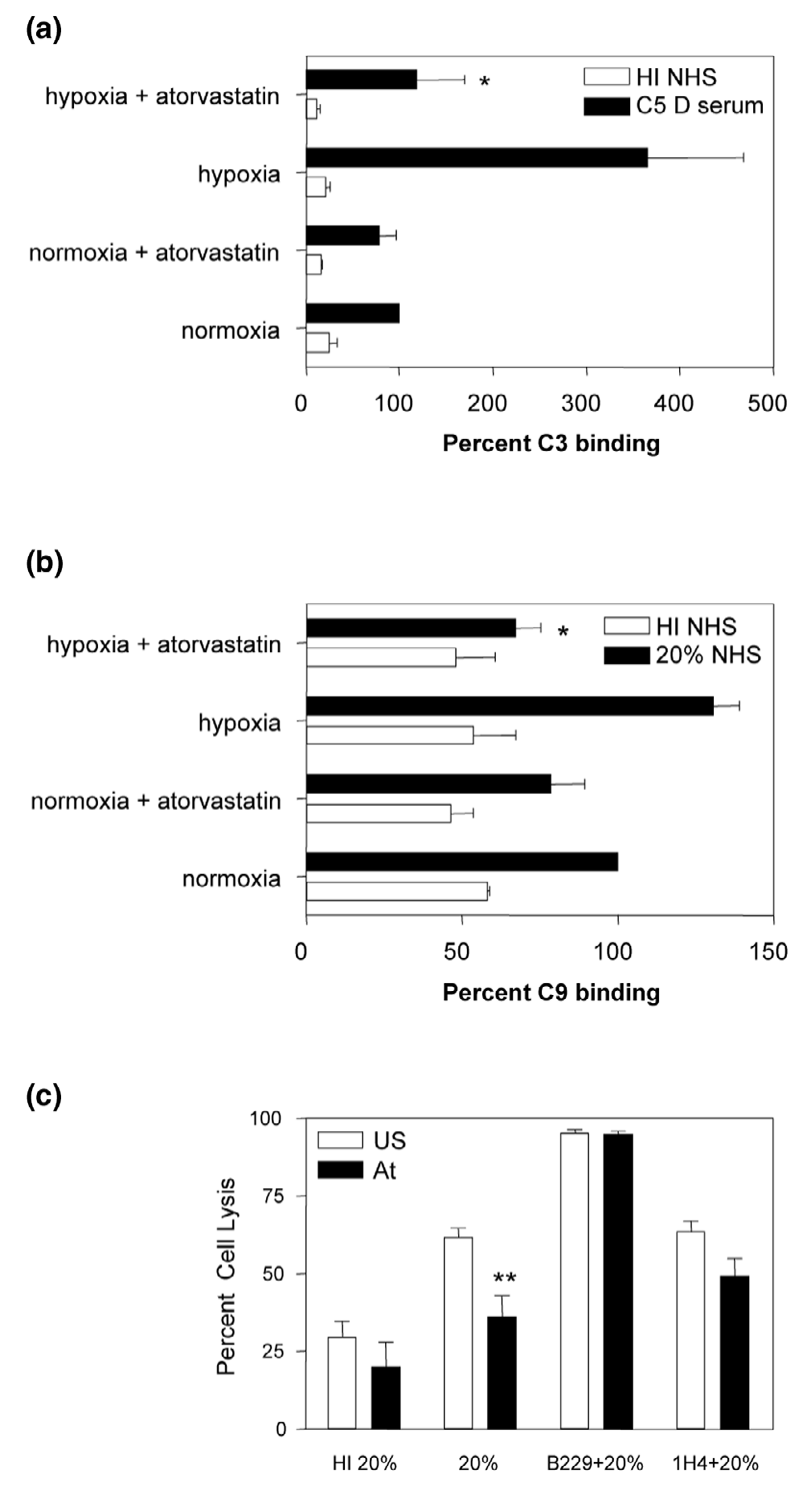
Atorvastatin-induced CD59 and decay-accelerating factor in hypoxia enhance endothelial cell cytoprotection. **(a) **Human umbilical vein endothelial cells (HUVEC) were cultured under normoxic or hypoxic conditions with and without atorvastatin (0.25 μM) for 48 hours followed by 3 hours reoxygenation. Harvested endothelial cells (EC) were incubated with 20% C5-deficient (C5 D) serum (filled bars) or heat-inactivated (HI) normal human serum (NHS) (open bars) for 2 hours. C3 binding was analysed by flow cytometry and results are expressed as the percentage of C3 binding relative to that on EC exposed to C5 D in normoxia (shown as 100%). *P < 0.05 (*n *= 4), difference between levels of cell surface C3 deposition on EC cultured under hypoxic conditions in the presence or absence of atorvastatin **(b) **HUVEC were cultured under normoxic or hypoxic conditions with and without atorvastatin (0.5 μM) for 48 hours followed by 3 hours of reoxygenation. C9 binding was analysed by flow cytometry following incubation with 20% NHS (filled bars) or HI serum (open bars). Results are expressed as the percentage of C9 binding relative to that on EC exposed to NHS in normoxia (shown as 100%). *P < 0.05 (*n *= 4), difference between statin-treated and untreated EC in hypoxia.**(c) **HUVEC were incubated in 1% O_2 _with or without atorvastatin (At) 0.5 μM for 48 hours followed by 3 hours of reoxygenation. EC were preincubated with the inhibitory mAbs Bric229 (CD59) and 1H4 (decay-accelerating factor) (20 μg/ml) or veronal buffered saline + 1% gelatin at 4°C. EC were then incubated with 20% rabbit serum or 20% HI rabbit serum at 37°C for 1 hour and propidium iodide (PI) was added prior to analysis by flow cytometry. The percentage EC lysis was calculated as the number of PI-positive cells expressed as a percentage of the total number of cells. **P < 0.001 (*n *= 4), difference between statin-treated and untreated EC.

To investigate the functional significance of the changes in CD59 and DAF expression, an *in vitro *model of complement activation induced by hypoxia–reoxygenation was used [13]. A fourfold increase in C3 deposition was detected on EC exposed to hypoxia–reoxygenation and 20% C5-deficient serum, when compared with those EC cultured under normoxic conditions (Figure 7a). The use of C5-deficient serum prevented the generation of the C5b-9 MAC, therefore facilitating investigation of the effects of DAF. Treatment of HUVEC with atorvastatin for 48 hours in hypoxia abolished C3 deposition on EC following reoxygenation (*P *< 0.05) (Figure 7a). The dependence upon complement activation was demonstrated by the lack of C3 deposition following exposure to heat-inactivated serum.

The functional effect of a change in CD59 expression was initially assessed by analysis of C9. A 40% increase in C9 binding was observed in HUVEC exposed to hypoxia–reoxygenation and 20% normal human serum, when compared with those HUVEC cultured in normoxia, and this was abrogated by pretreatment of EC with atorvastatin (Figure 7b) (*P *< 0.05).

A propidium iodide cell-lysis assay was used to quantify the outcome of complement activation on the EC surface. HUVEC cultured in 1% O_2 _were protected by atorvastatin against reoxygenation-induced complement-mediated EC lysis (*P *< 0.001) (Figure 7c). The importance of CD59 and DAF in this cytoprotection was confirmed using the neutralizing, noncomplement fixing mAbs BRIC 229 and 1H4, respectively. Statin-mediated protection was completely abolished by blockade of CD59 and was partially abolished following blockade of DAF (Figure 7c). Under hypoxic conditions, therefore, statins are capable of protecting EC against complement deposition through inhibition of both the C3 convertase and the MAC.

## Discussion

In the rheumatoid joint, synovial tissue hypertrophy and disorganized vasculature contribute to relative hypoperfusion and hypoxia, with consequent activation of HIF [1]. In addition, increased intra-articular pressure may cause capillary collapse on joint movement, resulting in repeated cycles of hypoxia–reoxygenation, chronic oxidative stress and enhanced local inflammation [1,2]. We used human EC, in an *in vitro *model system, to explore the effects of statins on complement activation in prolonged hypoxia, such as that found in the rheumatoid joint.

Complement activation plays an important role in the pathogenesis of RA and correlates with disease activity [8]. Immune complexes, rheumatoid factor and C-reactive protein OK may contribute to complement activation in the synovium [8]. In addition to this, *in vitro *studies with EC [13] suggest that cycles of hypoxia and reoxygenation within the synovium may also exacerbate complement activation. *In situ *analysis has demonstrated abundant local synthesis of C3, C3aR, C5aR and C5b-9 at distinct sites in the synovium [9], with C3 and C5b-9 expressed most strongly in the microvasculature, where C5b-9 deposition may result in endothelial injury [12]. Nucleated cells, however, are relatively resistant to lysis, and the effects of C5b-9 are more typically proinflammatory – with generation of reactive oxygen species, upregulation of E-selectin and intercellular adhesion molecule-1 on EC, and the release of soluble mediators including IL-8, MCP-1 and prostaglandin E_2 _[17,35,36], resulting in increased leukocyte recruitment in inflammatory arthritis [15]

The statins, principally used to control lipid levels, may also exert anti-inflammatory and immunomodulatory effects. Intriguingly, in two reports the statins displayed disease-modifying effects in rodent models of inflammatory arthritis [23,26], although a third study found no beneficial effect [25]. The Trial of Atorvastatin in Rheumatoid Arthritis [24] compared atorvastatin 40 mg daily with placebo, as an adjunct to existing antirheumatic therapy, and reported a significant improvement in the 28 joint disease activity score (DAS28) after 6 months. *In vivo *studies have demonstrated that statins reduce complement-dependent leukocyte migration [37] and that they may be protective against ischemia-reperfusion injury [38], in which complement activation plays an important role.

In view of the synergy observed between the actions of hypoxia and statins [30], we explored the effect of statins on the expression and function of membrane-bound CIP on EC, at levels of hypoxia consistent with those in the rheumatoid joint. A variety of different cell types is exposed to hypoxia and contributes to the pathogenesis of RA [6]. We chose to study vascular EC, as the endothelium is the portal of entry for leukocytes to the rheumatoid synovium and is particularly exposed to deposition of C3 and C5b-9 [9]. The concentrations of statins used were in the same range as those found to have effects on hypoxic human EC *in vitro *[30], and are close to those achieved in plasma following therapeutic dosing [39].

Treatment of HUVEC cultured in 1% O_2 _with atorvastatin, with lovastatin or with mevastatin resulted in upregulation of CD59, a response not seen in normoxia, where on occasion atorvastatin treatment reduced CD59 expression, although this did not reach significance. To our knowledge this is the most significant increase in CD59 protein expression recorded on primary human EC. Although CD59 is constitutively expressed on human vascular EC, we have failed to demonstrate significant upregulation in response to tumour necrosis factor alpha, interferon gamma, vascular endothelial growth factor or thrombin [27,40], and only a minimal change has been reported elsewhere in response to tumour necrosis factor alpha and IL-1β [41].

We have previously reported that, under normoxic conditions, statins upregulate EC DAF [21]. In the current study we show that hypoxia enhances atorvastatin-induced DAF expression, suggesting that hypoxia plays a permissive role in both CD59 and DAF upregulation by statins. Although the experiments described were performed with HUVEC, we have found comparable expression and regulation of CIP on both human arterial and microvascular EC [27,40].

Culture of EC in hypoxia (1% O_2_) is representative of the hypoxic conditions found within the rheumatoid joint and sufficient to activate HIF in EC [6]. We therefore sought to confirm the effects of hypoxia on CD59 and DAF expression, using agents that stabilize HIF in normoxia. Treatment of EC with atorvastatin and chemical mimetics of hypoxia demonstrated additive, and on occasion synergistic, increases in both CD59 and DAF. Treatment of cells with cobalt or iron chelators prevents von Hippel Lindau protein binding to HIF, which is required to target its destruction [42], thus mimicking hypoxia by stabilizing HIF in normoxic conditions. The permissive effect of both cobalt and iron chelation on DAF and CD59 expression suggests a role for HIF in the upregulation of DAF and CD59 by atorvastatin in hypoxia. The reported effects of statins on HIF expression are conflicting, however, with pravastatin increasing EC HIF-1 [43] and simvastatin reducing expression in coronary arteries [44]. Interestingly, although CD59 has not been shown to be a hypoxia-responsive gene, microarray analysis of von Hippal Lindau regulated genes revealed CD59 to be a von Hippal Lindau target [45].

CD59 upregulation by atorvastatin in hypoxia was dependent upon increased steady-state mRNA, with maximal induction at 8 hours returning to baseline 16 hours post-treatment. We did not detect any effect of atorvastatin on endothelial nitric oxide synthase mRNA stability. A small increase in CD59 mRNA was also seen in normoxic conditions following 8 hours of treatment with atorvastatin, with a further increase under hypoxic conditions. Of note, despite a small increase in mRNA, no significant change in CD59 surface protein expression was detectable following treatment with atorvastatin in normoxia, raising the possibility that increased expression in hypoxic conditions reflects an additional effect of hypoxia that facilitates CD59 translation or surface expression. It is noteworthy that the upregulation by statins and hypoxia of another glycosylphosphatidylinositol-anchored molecule, ecto-5' -nucleotidase (CD73), relies on reduced endocytosis, as a result of alteration in the membrane fatty acid content under hypoxic conditions and of statin-mediated inhibition of Rho [30].

Statins also inhibit geranylgeranylation and farnesylation through the inhibition of HMG-CoA reductase, therefore preventing the post-translational modification of the GTP-binding proteins Rho, Rac and Ras. This results in anti-inflammatory effects including the downregulation of NF-κB activity [46], the stabilization of endothelial nitric oxide synthase mRNA and increased NO biosynthesis [33]. As many of the cytoprotective effects of statins in hypoxia are NO-dependent, we explored the role of NO using L-NMMA and L-NAME, which significantly inhibited upregulation of CD59 in hypoxia. We also demonstrated that the regulation of CD59 by statins in hypoxia was inhibited by mevalonate and geranylgeraniol, confirming a role for inhibition of HMG-CoA reductase and geranylgeranylation, respectively. Furthermore, the failure of squalene to influence the response suggested that the mechanism underlying the actions of the statins was cholesterol independent. Although the effect of statins on farnesylation was not studied, we have previously reported that inclusion of farnesylpyrophosphate does not inhibit statin-induced DAF expression [21], and likewise that geranylgeranyl pyrophosphate and not farnesylpyrophosphate inhibit statin-induced changes in NO bioavailability [33].

Notwithstanding this information, the precise mechanism underlying the effects of hypoxia and NO in statin-induced CD59 expression remains to be fully determined. We have previously shown that statin-induced DAF expression in normoxia is independent of NO [21], suggesting that a distinct additional mechanism is activated by the combination of statins and the hypoxic microenvironment, resulting in induction of CD59 and enhanced DAF upregulation. The involvement of NO may reflect its ability to activate protein kinase C epsilon [47], a protein kinase C isoenzyme capable of regulating DAF expression [48]. Furthermore, NO is reported to inhibit phosphatidylinositol-specific phospholipase C, thus reducing shedding of glycosylphosphatidylinositol-anchored proteins such as CD59 and DAF [49]. Additional mechanisms are also likely to be important and dependent upon the redox status of EC. Other cytoprotective molecules such as adenosine may therefore contribute, as HUVEC exposed to hypoxia and statins upregulate CD73 expression, releasing adenosine [22], which can induce NO synthesis.

CD59 appears to play an important role in the joint and its expression is reported to be reduced in rheumatoid synovium when compared with noninflamed tissue [11]. The hypothesis that CD59 deficiency may contribute to synovial inflammation in RA is supported by the report that deletion of CD59a, the murine homologue of human CD59, increased disease severity in an antigen-induced arthritis model, a phenotype that was reversed by recombinant membrane-targeted CD59 [20]. These studies clearly implicate C5b-9 as pathogenic and CD59 as a protective factor in murine models of RA. Complement activation therefore represents an attractive therapeutic target in RA. Various approaches are effective in rodent models, including treatment with an anti-C5 mAb [50], soluble complement receptor-1 and a DAF-Ig fusion protein [51,52]. Moreover, C5-deficiency protects susceptible mice (DBA/1LacJ) against CIA [53]. Although data from human studies are limited, anti-C5 mAb therapy has been reported safe and effective in RA [54].

To explore the functional relevance of statin-induced CIP expression we utilized a hypoxia-reoxygenation model [36]. The increased expression of CD59 and DAF, induced by statins under hypoxic conditions, significantly reduced complement activation and cell lysis following hypoxia–reoxygenation. The anti-inflammatory effects of statins in RA are likely to be multifactorial and include effects on T cells and monocyte/macrophage function, on proinflammatory cytokine release, on leukocyte trafficking and on generation of reactive oxygen species [24]. The results herein suggest that modulation of complement activation, through induction of membrane-bound CIP, should be added to this list. In particular, statin-induced CD59 expression would act to reverse the deficiency seen in RA [11] and would minimize the proinflammatory actions of C5b-9, which are not only confined to the vasculature but also affect synovial cells, resulting in the release of proinflammatory mediators [15].

Although the role of statins in RA therapy remains to be determined, they represent an attractive option. RA is associated with chronic endothelial dysfunction and a twofold to threefold increase in the risk of myocardial infarction. The results of the Trial of Atorvastatin in Rheumatoid Arthritis study show that atorvastatin significantly reduces levels of low-density lipoprotein-cholesterol and triglyceride in RA, while also exerting measurable disease-modifying effects – suggesting that statins offer both vascular protection and adjunctive immunomodulatory potential in RA [24]. Recognizing the preliminary nature of the clinical data supporting a disease-modifying effect for statins in RA and the need for *in vivo *confirmation of our findings, we propose that the ability of statins to significantly increase expression of membrane-bound CIP on vascular EC under hypoxic conditions may contribute to an anti-inflammatory action of statins in RA. The combined effects of DAF, at the level of C3 and C5 convertases, and of CD59 inhibiting the terminal attack complex has the potential to exert anti-inflammatory and vasculoprotective effects, both in the synovium and at sites of atherogenesis.

## Conclusion

We have identified a novel mechanism by which statins protect the vascular endothelium against complement deposition following hypoxia–reoxygenation, through increased expression of CD59, via an NO-dependent and lipid-independent pathway. This, combined with statin-induced DAF upregulation, may represent an important contributory mechanism by which statin therapy can provide both anti-inflammatory and anti-atherogenic effects in RA.

## Abbreviations

CIP = complement-inhibitory protein; CoCl_2 _= cobalt chloride; DAF = decay-accelerating factor; DFO = desferrioxamine; EC = endothelial cells; HIF = hypoxia-inducible factor; HMG-CoA = 3-hydroxy-3-methylglutaryl coenzyme A; HUVEC = human umbilical vein endothelial cells; IL = interleukin; L-NAME = *N*^G^-nitro-L-arginine methyl ester; L-NMMA = *N*^G^-monomethyl-L-arginine; mAb = monoclonal antibody; MAC = membrane attack complex; MCP = membrane cofactor protein; NF = nuclear factor; NO = nitric oxide; RA = rheumatoid arthritis; PCR = polymerase chain reaction; RFI = relative fluorescence intensity; VBSG = veronal buffered saline/1% gelatin.

## Competing interests

PHM is a shareholder, founder, consultant and director of ReOx Ltd.

## Authors' contributions

ARK performed endothelial cell isolation, culture and stimulation, flow-cytometric and northern analysis, carried out the complement functional assays, and participated in study conception and design and drafting of the manuscript, with the assistance of coauthors. RS contributed to generation of endothelial cell cultures and flow-cytometric analysis. EAL was involved in endothelial cell isolation, northern analysis and study design. PHM participated in the design of the study and supervised experiments performed in the hypoxic chamber. MJ performed the real-time PCR analyses and SKH ran the western blots and supervised experiments performed in the hypoxic chamber. DOH participated in the study design and conduct and in editing the manuscript. JCM conceived of the study, participated in its design and coordination, and participated in drafting and editing the manuscript. All authors read and approved the final version of the manuscript.

## References

[B1] Distler JH, Wenger RH, Gassmann M, Kurowska M, Hirth A, Gay S, Distler O (2004). Physiologic responses to hypoxia and implications for hypoxia-inducible factors in the pathogenesis of rheumatoid arthritis. Arthritis Rheum.

[B2] Taylor PC, Sivakumar B (2005). Hypoxia and angiogenesis in rheumatoid arthritis. Curr Opin Rheumatol.

[B3] Stevens CR, Blake DR, Merry P, Revell PA, Levick JR (1991). A comparative study by morphometry of the microvasculature in normal and rheumatoid synovium. Arthritis Rheum.

[B4] Cernanec J, Guilak F, Weinberg JB, Pisetsky DS, Fermor B (2002). Influence of hypoxia and reoxygenation on cytokine-induced production of proinflammatory mediators in articular cartilage. Arthritis Rheum.

[B5] Demasi M, Cleland LG, Cook-Johnson RJ, James MJ (2004). Effects of hypoxia on the expression and activity of cyclooxygenase 2 in fibroblast-like synoviocytes: interactions with monocyte-derived soluble mediators. Arthritis Rheum.

[B6] Giatromanolaki A, Sivridis E, Maltezos E, Athanassou N, Papazoglou D, Gatter KC, Harris AL, Koukourakis MI (2003). Upregulated hypoxia inducible factor-1a and -2a pathway in rheumatoid arthritis and osteoarthritis. Arthritis Res Ther.

[B7] Hollander AP, Corke KP, Freemont AJ, Lewis CE (2001). Expression of hypoxia-inducible factor 1alpha by macrophages in the rheumatoid synovium: implications for targeting of therapeutic genes to the inflamed joint. Arthritis Rheum.

[B8] Molenaar ET, Voskuyl AE, Familian A, van Mierlo GJ, Dijkmans BA, Hack CE (2001). Complement activation in patients with rheumatoid arthritis mediated in part by C-reactive protein. Arthritis Rheum.

[B9] Neumann E, Barnum SR, Tarner IH, Echols J, Fleck M, Judex M, Kullmann F, Mountz JD, Scholmerich J, Gay S (2002). Local production of complement proteins in rheumatoid arthritis synovium. Arthritis Rheum.

[B10] Tarkowski A, Trollmo C, Seifert PS, Hansson GK (1992). Expression of decay-accelerating factor on synovial lining cells in inflammatory and degenerative arthritides. Rheumatol Int.

[B11] Konttinen YT, Ceponis A, Meri S, Vuorikoski A, Kortekangas P, Sorsa T, Sukura A, Santavirta S (1996). Complement in acute and chronic arthritides: assessment of C3c, C9, and protectin (CD59) in synovial membrane. Ann Rheum Dis.

[B12] Kato H, Yamakawa M, Ogino T (2000). Complement mediated vascular endothelial injury in rheumatoid nodules: a histopathological and immunohistochemical study. J Rheumatol.

[B13] Collard CD, Vakeva A, Bukusoglu C, Zund G, Sperati CJ, Colgan SP, Stahl GL (1997). Reoxygenation of hypoxic human umbilical vein endothelial cells activates the classic complement pathway. Circulation.

[B14] Tramontini NL, Kuipers PJ, Huber CM, Murphy K, Naylor KB, Broady AJ, Kilgore KS (2002). Modulation of leukocyte recruitment and IL-8 expression by the membrane attack complex of complement (C5b-9) in a rabbit model of antigen-induced arthritis. Inflammation.

[B15] Daniels RH, Houston WA, Petersen MM, Williams JD, Williams BD, Morgan BP (1990). Stimulation of human rheumatoid synovial cells by non-lethal complement membrane attack. Immunology.

[B16] Kilgore KS, Shen JP, Miller BF, Ward PA, Warren JS (1995). Enhancement by the complement membrane attack complex of tumor necrosis factor-alpha-induced endothelial cell expression of E-selectin and ICAM-1. J Immunol.

[B17] Kilgore KS, Flory CM, Miller BF, Evans VM, Warren JS (1996). The membrane attack complex of complement induces interleukin-8 and monocyte chemoattractant protein-1 secretion from human umbilical vein endothelial cells. Am J Pathol.

[B18] Liszewski MK, Farries TC, Lublin DM, Rooney IA, Atkinson JP (1996). Control of the complement system. Adv Immunol.

[B19] Mizuno M, Nishikawa K, Spiller OB, Morgan BP, Okada N, Okada H, Matsuo S (2001). Membrane complement regulators protect against the development of type II collagen-induced arthritis in rats. Arthritis Rheum.

[B20] Williams AS, Mizuno M, Richards PJ, Holt DS, Morgan BP (2004). Deletion of the gene encoding CD59a in mice increases disease severity in a murine model of rheumatoid arthritis. Arthritis Rheum.

[B21] Mason JC, Ahmed Z, Mankoff R, Lidington EA, Ahmad S, Bhatia V, Kinderlerer A, Randi AM, Haskard DO (2002). Statin-induced expression of decay-accelerating factor protects vascular endothelium against complement-mediated injury. Circ Res.

[B22] Ledoux S, Runembert I, Koumanov K, Michel JB, Trugnan G, Friedlander G (2003). Hypoxia enhances ecto-5'-nucleotidase activity and cell surface expression in endothelial cells: role of membrane lipids. Circ Res.

[B23] Leung BP, Sattar N, Crilly A, Prach M, McCarey DW, Payne H, Madhok R, Campbell C, Gracie JA, Liew FY (2003). A novel anti-inflammatory role for simvastatin in inflammatory arthritis. J Immunol.

[B24] McCarey DW, McInnes IB, Madhok R, Hampson R, Scherbakova O, Ford I, Capell HA, Sattar N (2004). Trial of Atorvastatin in Rheumatoid Arthritis (TARA): double-blind, randomised placebo-controlled trial. Lancet.

[B25] Palmer G, Chobaz V, Talabot-Ayer D, Taylor S, So A, Gabay C, Busso N (2004). Assessment of the efficacy of different statins in murine collagen-induced arthritis. Arthritis Rheum.

[B26] Barsante MM, Roffe E, Yokoro CM, Tafuri WL, Souza DG, Pinho V, Castro MSD, Teixeira MM (2005). Anti-inflammatory and analgesic effects of atorvastatin in a rat model of adjuvant-induced arthritis. Eur J Pharmacol.

[B27] Mason JC, Yarwood H, Sugars K, Morgan BP, Davies KA, Haskard DO (1999). Induction of decay-accelerating factor by cytokines or the membrane-attack complex protects vascular endothelial cells against complement deposition. Blood.

[B28] Coyne KE, Hall SE, Thompson S, Arce MA, Kinoshita T, Fujita M, Anstee DJ, Rosse WF, Lublin DM (1992). Mapping of epitopes, glycosylation sites, and complement regulatory domains in human decay accelerating factor. J Immunol.

[B29] Morgan BP (1992). Isolation and characterization of the complement-inhibiting protein CD59 antigen from platelet membranes. Biochem J.

[B30] Ledoux S, Laouari D, Essig M, Runembert I, Trugnan G, Michel JB, Friedlander G (2002). Lovastatin enhances ecto-5'-nucleotidase activity and cell surface expression in endothelial cells: implication of rho-family GTPases. Circ Res.

[B31] Graham CH, Fitzpatrick TE, McCrae KR (1998). Hypoxia stimulates urokinase receptor expression through a heme protein-dependent pathway. Blood.

[B32] Laufs U, Fata VL, Liao JK (1997). Inhibition of 3-hydroxy-3-methylglutaryl (HMG)-CoA reductase blocks hypoxia-mediated down-regulation of endothelial nitric oxide synthase. J Biol Chem.

[B33] Laufs U, Liao JK (1998). Post-transcriptional regulation of endothelial nitric oxide synthase mRNA stability by Rho GTPase. J Biol Chem.

[B34] Sohn HY, Krotz F, Gloe T, Keller M, Theisen K, Klauss V, Pohl U (2003). Differential regulation of xanthine and NAD(P)H oxidase by hypoxia in human umbilical vein endothelial cells. Role of nitric oxide and adenosine. Cardiovasc Res.

[B35] Tedesco F, Pausa M, Nardon E, Introna M, Mantovani A, Dobrina A (1997). The cytolytically inactive terminal complement complex activates endothelial cells to express adhesion molecules and tissue factor procoagulant activity. J Exp Med.

[B36] Collard CD, Agah A, Reenstra W, Buras J, Stahl GL (1999). Endothelial nuclear factor-kappaB translocation and vascular cell adhesion molecule-1 induction by complement: inhibition with anti-human C5 therapy or cGMP analogues. Arterioscler Thromb Vasc Biol.

[B37] Fischetti F, Carretta R, Borotto G, Durigutto P, Bulla R, Meroni PL, Tedesco F (2004). Fluvastatin treatment inhibits leucocyte adhesion and extravasation in models of complement-mediated acute inflammation. Clin Exp Immunol.

[B38] Di Napoli P, Taccardi AA, Grilli A, De Lutiis MA, Barsotti A, Felaco M, De Caterina R (2005). Chronic treatment with rosuvastatin modulates nitric oxide synthase expression and reduces ischemia-reperfusion injury in rat hearts. Cardiovasc Res.

[B39] Cilla DD, Whitfield LR, Gibson DM, Sedman AJ, Posvar EL (1996). Multiple-dose pharmacokinetics, pharmacodynamics, and safety of atorvastatin, an inhibitor of HMG-CoA reductase, in healthy subjects. Clin Pharmacol Ther.

[B40] Mason JC, Lidington EA, Yarwood H, Lublin DM, Haskard DO (2001). Induction of endothelial cell decay-accelerating factor by vascular endothelial growth factor: a mechanism for cytoprotection against complement-mediated injury during inflammatory angiogenesis. Arthritis Rheum.

[B41] Moutabarrik A, Nakanishi I, Namiki M, Hara T, Matsumoto M, Ishibashi M, Okuyama A, Zaid D, Seya T (1993). Cytokine-mediated regulation of the surface expression of complement regulatory proteins, CD46(MCP), CD55(DAF), and CD59 on human vascular endothelial cells. Lymphokine Cytokine Res.

[B42] Hon WC, Wilson MI, Harlos K, Claridge TD, Schofield CJ, Pugh CW, Maxwell PH, Ratcliffe PJ, Stuart DI, Jones EY (2002). Structural basis for the recognition of hydroxyproline in HIF-1 alpha by pVHL. Nature.

[B43] Chen SD, Hu CJ, Yang DI, Nassief A, Chen H, Yin K, Xu J, Hsu CY (2005). Pravastatin attenuates ceramide-induced cytotoxicity in mouse cerebral endothelial cells with HIF-1 activation and VEGF upregulation. Ann NY Acad Sci.

[B44] Wilson SH, Herrmann J, Lerman LO, Holmes DR, Napoli C, Ritman EL, Lerman A (2002). Simvastatin preserves the structure of coronary adventitial vasa vasorum in experimental hypercholesterolemia independent of lipid lowering. Circulation.

[B45] Zatyka M, da Silva NF, Clifford SC, Morris MR, Wiesener MS, Eckardt KU, Houlston RS, Richards FM, Latif F, Maher ER (2002). Identification of cyclin D_1 _and other novel targets for the von Hippel-Lindau tumor suppressor gene by expression array analysis and investigation of cyclin D_1 _genotype as a modifier in von Hippel-Lindau disease. Cancer Res.

[B46] Dichtl W, Dulak J, Frick M, Alber HF, Schwarzacher SP, Ares MP, Nilsson J, Pachinger O, Weidinger F (2003). HMG-CoA reductase inhibitors regulate inflammatory transcription factors in human endothelial and vascular smooth muscle cells. Arterioscler Thromb Vasc Biol.

[B47] Balafanova Z, Bolli R, Zhang J, Zheng Y, Pass JM, Bhatnagar A, Tang XL, Wang O, Cardwell E, Ping P (2002). Nitric oxide (NO) induces nitration of protein kinase Cε (PKCε), facilitating PKCε translocation via enhanced PKCε–RACK2 interactions. a novel mechanism of NO-triggered activation of PKCε. J Biol Chem.

[B48] Mason JC, Steinberg R, Lidington EA, Kinderlerer AR, Ohba M, Haskard DO (2004). Decay-accelerating factor induction on vascular endothelium by vascular endothelial growth factor (VEGF) is mediated via a VEGF receptor-2 (VEGF-R2)- and protein kinase C-a/ε (PKCa/ε)-dependent cytoprotective signaling pathway and is inhibited by cyclosporin A. J Biol Chem.

[B49] Park SW, Yoon HJ, Lee HB, Hooper NM, Park HS (2002). Nitric oxide inhibits the shedding of the glycosylphosphatidylinositol-anchored dipeptidase from porcine renal proximal tubules. Biochem J.

[B50] Wang Y, Rollins SA, Madri JA, Matis LA (1995). Anti-C5 monoclonal antibody therapy prevents collagen-induced arthritis and ameliorates established disease. Proc Natl Acad Sci USA.

[B51] Goodfellow RM, Williams AS, Levin JL, Williams BD, Morgan BP (1997). Local therapy with soluble complement receptor 1 (sCR1) suppresses inflammation in rat mono-articular arthritis. Clin Exp Immunol.

[B52] Harris CL, Williams AS, Linton SM, Morgan BP (2002). Coupling complement regulators to immunoglobulin domains generates effective anti-complement reagents with extended half-life *in vivo*. Clin Exp Immunol.

[B53] Wang Y, Kristan J, Hao L, Lenkoski CS, Shen Y, Matis LA (2000). A role for complement in antibody-mediated inflammation: C5-deficient DBA/1 mice are resistant to collagen-induced arthritis. J Immunol.

[B54] Tesser J, Kivitz A, Fleischmann R, Mojcik CF, Bombara M, Burch F (2001). Safety and efficacy of the humanized anti-C5 antibody h5G1.1 in patients with rheumatoid arthritis [abstract]. Arthritis Rheum.

